# Reduced STAG2 expression in myelodysplastic neoplasms and acute myeloid leukemia myelodysplasia-related: a potential biomarker associated with aneuploidy and disease progression

**DOI:** 10.3389/fcell.2026.1731983

**Published:** 2026-03-20

**Authors:** Beatriz Ferreira da Silva, Nina Carrossini Bastos, Tatiana Fonseca Alvarenga, Rafael Mina Piergiorge, Verônica Goulart Moreira, Ana Paula Bueno, Cristiane Bedran Milito, Cíntia Barros Santos-Rebouças, Teresa De Souza Fernandez

**Affiliations:** 1 Cytogenetic Laboratory, Cell and Gene Therapy Program, Instituto Nacional de Câncer (INCA), Rio de Janeiro, Rio de Janeiro, Brazil; 2 Pathology Division (DIPAT), Instituto Nacional de Câncer (INCA), Rio de Janeiro, Rio de Janeiro, Brazil; 3 Human Genetics Laboratory (ServGen), Department of Genetics, Institute of Biology Roberto Alcantara Gomes, Rio de Janeiro State University, Rio de Janeiro, Brazil; 4 Faculdade de Medicina, Instituto de Pediatria e Puericultura Martagão Gesteira, Universidade Federal do Rio de Janeiro (UFRJ), Rio de Janeiro, Rio de Janeiro, Brazil

**Keywords:** AML-MR, aneuploidy, biomarker, leukemic evolution, myelodysplastic neoplasms, STAG2

## Abstract

**Background:**

Myelodysplastic neoplasms (MDS) are a clonal hematopoietic stem cell disease with an increased risk of progression to acute myeloid leukemia (AML). Cytogenetic abnormalities, mainly aneuploidies, occur in 40%–60% of cases and are critical for MDS diagnosis and prognosis. Nevertheless, the MDS heterogeneity highlights the ongoing need for additional studies regarding mechanisms driving its development and progression, contributing to the identification of biomarkers. In this context, *STAG2*, a key component of the cohesin complex, has been implicated in the pathogenesis of MDS. However, the expression of the STAG2 protein and its role in MDS biology remain unexplored. Thus, this study aimed to analyze the STAG2 protein expression profile in MDS patients, its association with karyotypes, evolution to AML, and its potential as a prognosis biomarker.

**Methods:**

STAG2 expression was analyzed by immunohistochemistry in bone marrow biopsies from 97 MDS patients (24 pediatric, 73 adult) and 20 controls (10 pediatric, 10 adult). Cytogenetic analysis was performed by G-banding and fluorescence *in situ* hybridization (FISH). Protein–protein interaction (PPI) networks were conducted using the STRING database, and functional enrichment analyses were performed using Gene Ontology (GO), Kyoto Encyclopedia of Genes and Genomes (KEGG), Reactome, and Transcription factors PPIs (TFPPI) databases.

**Results:**

Reduced STAG2 expression was detected in 67% (65/97) of MDS cases, with 51.5% exhibiting intermediate and 15% low expression. Abnormal karyotypes were found in 41% (40/97) of patients, among them STAG2 reduction was present in 65% (13/20) with structural alterations, 69% (9/13) with aneuploidies, and 86% (6/7) with both. Reduced STAG2 expression was particularly associated with complex karyotypes (86%) and trisomy of chromosome 8 (83%). Bioinformatic analysis showed that STAG2 interactors were enriched in pathways related to mitotic regulation, chromatin and epigenetic control, and 3D genome organization.

**Conclusion:**

Our findings suggest that reduced STAG2 protein expression plays a relevant role in MDS pathogenesis. Immunohistochemical assessment of STAG2 may be incorporated into the clinical workflows as a potential diagnostic and prognostic biomarker, as well as indicate therapeutic targets, particularly in association with complex karyotypes and trisomy 8.

## 1 Introduction

Myelodysplastic neoplasms (MDS) are a clonal hematopoietic stem cell disease that can manifest across all age groups; however, its incidence increases with advancing age. In this sense, it represents the most common hematologic malignancy in the elderly population and is rare in pediatric patients (Babcock et al., 2023; Rotter et al., 2023). MDS is characterized by bone marrow dysplasias, which compromise the effectiveness of hematopoiesis, leading to peripheral blood cytopenias (Rotter et al., 2023; Sekeres and Taylor, 2022). The clinical course of MDS is notably heterogeneous, ranging from indolent forms with slow progression to aggressive subtypes that rapidly progress to acute myeloid leukemia (AML) (Gangat et al., 2016).

Cytogenetic abnormalities are present in 40%–60% of cases, commonly manifesting as aneuploidies, such as monosomy 7 and trisomy 8 (Sekeres and Taylor, 2022; Gangat et al., 2016; Babcock et al., 2023). Cytogenetics has been an important tool in elucidating the pathogenesis of MDS, offering critical insights into disease mechanisms while serving as a fundamental component in both diagnosis and prognostic risk stratification (Sekeres and Taylor, 2022; Gangat et al., 2016; Babcock et al., 2023; Greenberg et al., 2012). Despite these advances, the considerable heterogeneity of MDS underscores the ongoing need for additional studies regarding mechanisms driving its development and progression. Therefore, contributing to the identification of biomarkers.

Analyses of the genomic landscape in adult patients with MDS have identified mutations in genes involved in several pathways, such as splicing, DNA methylation, cell signaling, and chromatin regulation (Bernard et al., 2022; Bersanelli et al., 2021; Shreve and Nazha, 2020; Saygin and Godley, 2021). Among these, altered chromatin organization has emerged as a central mechanism in the MDS pathogenesis, especially due to alterations in *STAG2*, which is the most affected cohesin complex component (Xu and Viny, 2024).

STAG2, also known as the cohesin subunit SA-2, is a fundamental component of the cohesin complex and the most abundant subunit in humans (Brooker and Berkowitz, 2014; Golov and Gavrilov, 2024). Alterations in components of this complex were first described in cancer patients about 10–15 years ago (Solomon et al., 2011). However, this association gained greater relevance when recurrent *STAG2* mutations were linked to aneuploidy in multiple tumor cell lineages (Solomon et al., 2011; Solomon et al., 2014). Since then, subsequent research has expanded the role of the cohesin complex beyond sister chromatid cohesion to include the maintenance of three-dimensional (3D) genome architecture, gene expression regulation, and DNA repair (Waldman, 2020; Di Nardo et al., 2022). Consequently, dysfunction of cohesin complex components has been directly implicated in genomic stability, a central hallmark of cancer (Hanahan, 2022).

Regarding the protein STAG2, its expression in samples of MDS patients has not yet been evaluated, regardless of age group. The analysis of protein expression by immunohistochemistry is important in the study of neoplasms. This analysis allows a direct visualization and spatial localization of the final product of gene expression, in the tissue studied, providing important information on both distribution and abundance of the protein in complex and heterogeneous tissue contexts (Kabiraj and Tomar Bhattacharya, 2015; Kremer et al., 2005; Taube et al., 2020). Thus, this study aimed to analyze the STAG2 protein expression profile by immunohistochemistry in pediatric and adult MDS patients, its association with karyotypes, evolution to AML, and its potential as a prognosis biomarker.

## 2 Materials and methods

### 2.1 Patients

This study was conducted using bone marrow tissue samples fixed in formalin and embedded in paraffin (bone marrow biopsy) with a date compatible with the cytogenetic analysis (diagnosis) of 97 MDS patients (24 pediatric and 73 adult). As controls for STAG2 expression, 20 bone marrow biopsy samples, 10 pediatric and 10 adult, from oncologic patients were used. These bone marrow samples were free from neoplasia, and the patients hadn't received chemotherapeutic treatment. The bone marrow samples of patients with MDS were obtained from the Bone Marrow Transplant Center (CEMO), Hematology Service at the National Cancer Institute (INCA), Rio de Janeiro, Brazil, and the Martagão Gesteira Pediatric and Puericulture Institute (IPPMG) at the Federal University of Rio de Janeiro (UFRJ), Rio de Janeiro, Brazil. Inclusion criteria comprised patients with primary MDS (pediatric, until 18 years of age, and adults, over 18 years of age), confirmed through clinical, morphological, cytogenetic, and immunophenotypic analyses. Exclusion criteria included patients with secondary MDS associated with the treatment of another primary neoplasm and MDS with genetic predisposition syndromes. Diagnosis was carried out by hematologists at the original institutions. The classification followed the recent recommendations of the World Health Organization (WHO) (Khoury et al., 2022). This study also included patients with 20%–28% blasts in the bone marrow, who fulfilled immunophenotypic, cytogenetic, and morphological features consistent with MDS classified as AML, myelodysplasia-related (AML-MR) (Khoury et al., 2022). This study was approved by the Ethics and Research Committee of the National Cancer Institute (reference number # 3401739) and following the Declaration of Helsinki.

### 2.2 Cytogenetic analysis

The cytogenetic analyses were carried out through G-banding according to standard cytogenetic methods on bone marrow samples of 97 MDS patients. Chromosome analyses were done according to the International System for Human Cytogenomic Nomenclature (ISCN, 2020) (McGowan-Jordan et al., 2020). Fluorescence *in situ* hybridization (FISH) analyses were performed as a complementary technique for 72 cases in which G-banding suggested an alteration, but the mitotic index was low, to confirm chromosomal deletions, characterize the breakpoint, and identify the gene involved. A FISH panel with probes for MDS was also used on samples with normal karyotypes to confirm the absence of specific alterations in a larger number of analyzed cells. The probes used were: -7/del (7q) (D7S486 spectrum orange/CEP7 spectrum green), +8 (LSI cMYC, spectrum orange), del (11) (q23) (LSI MLL dual-color break-apart rearrangement probe), del (17) (p13) (LSI p53, spectrum orange), del (5) (q31) (LSI CSF1R “spectrum orange”/LSID5S23:D5S721 spectrum green) (Vyses, Abbott Laboratories, United States). The slides were prepared from the cytogenetic cultures according to the manufacturer’s protocols.

### 2.3 Analysis of STAG2 expression by immunohistochemistry

Immunohistochemical staining was performed using a validated STAG2 antibody (PA5-78340, ThermoFisher Scientific, United States). Bone marrow tissue blocks were sectioned at 3 µm and positioned on marked slides (Histosilane, Inopat). After fixation at 60 °C for 16 h, sections were deparaffinized through xylene and rehydrated through graded ethanol washes, followed by antigen retrieval in Trilogy buffer solution (Cell Marque) at 98 °C for 30 min. Endogenous peroxidase and nonspecific binding were blocked using the NovoLink Max Polymer Detection Kit (Leica Microsystems). The primary antibody was applied to the sections and incubated overnight at 4 °C. After staining, sections were counterstained with Harris hematoxylin, dehydrated, and soaked in xylene. Slides were then mounted using Erv-Mount medium (EasyPath).

Two pathologists independently analyzed the slides. STAG2 expression was scored through a semiquantitative method (Shulman et al., 2022), combining the percentage of positive cells and staining intensity. The percentage of positive cells was categorized as follows: <30% (one point), 30%–75% (two points), and >75% (three points). Staining intensity was also graded as weak (one point), moderate (two points), or strong (three points).

Patients were classified into three categories based on the total score: those scoring 2 points were considered to have low STAG2 expression, scores between 3 and 5 indicated intermediate expression, and 6 indicated high STAG2 expression. The STAG2 expression pattern was analyzed according to the cytogenetic profile, subtypes, and disease progression.

### 2.4 Bioinformatics analysis

To further explore the relationship among STAG2, MDS, AML, and abnormal karyotypes, protein–protein interaction (PPI) networks were retrieved from Search Tool for the Retrieval of Interacting Genes/Proteins (STRING, version 12.0). Independent searches were carried out for “myelodysplastic syndrome” (MDS, DOID:0050908) and “acute myeloid leukemia” (AML, DOID:9119) using a high confidence interaction score threshold of 0.7, with no more than 50 interactors in the first shell and no more than 5 interactors in the second shell. Active interaction sources included text mining, experimental data, curated databases, co-expression, neighborhood, gene fusion, and co-occurrence. STAG2 interactions were retrieved with the highest confidence score (0.9), with all interactors in the first and second shells.

Proteins obtained from STRING were annotated using BioMart (Ensembl Genes 115, *Homo sapiens*, GRCh38. p14) to retrieve the corresponding gene names, genomic coordinates, and chromosome location. Specific attention was given to proteins directly or indirectly interacting with STAG2, for which a circular genomic plot was generated to visualize their chromosomal distribution. MDS and AML-related proteins were assigned to STAG2-associated interactors identified in the circular plot. Besides, functional enrichment analysis through Gene Ontology (GO), Kyoto Encyclopedia of Genes and Genomes (KEGG), Reactome, and Transcription factors PPIs (TFPPI) databases was performed for STAG2 direct and indirect interactors.

## 3 Results

### 3.1 Clinical and cytogenetic characteristics

Of the 24 pediatric patients, the initial subtype, childhood MDS with low blasts (cMDS-LB), was the most prevalent, observed in 92% (22/24) of patients. Childhood MDS with increased blasts (cMDS-IB) was observed in 4% (1/24) and AML-MR in 4% (1/24). Concerning adult patients, the initial subtypes were also the most prevalent (66%), with 56% (41/73) as MDS with low blasts (MDS-LB) and 10% (7/73) as MDS hypoplastic (MDS-h). MDS with increased blasts 1 (MDS-IB-1) and MDS with increased blasts 2 (MDS-IB-2) were observed in 13% (10/73) and 16% (12/73), respectively. AML-MR was observed in 4% (3/73) (Table 1).

**TABLE 1:** Clinical and cytogenetics characteristics of the MDS patients.

Patients	Pediatric	Adults	Total
Gender			
Male	17 (71%)	30 (41%)	47 (48,5%)
Female	7 (29%)	43 (59%)	50 (51,5%)
Mean age (range)	9.7 (7 months - 18 years)	52 (20–85 years)	47.7 (7 months - 85 years)
Subtypes			
Initial (MDS-LB, cMDS-LB MDS-h)	22 (92%)	48 (66%)	70 (72%)
Advanced (cMDS-IB, MDS-IB-1, MDS-IB-2) and (AML-MR)	2 (8%)	25 (34%)	27 (28%)
Cytogenetics			
Normal	18 (75%)	39 (53%)	57 (59%)
Abnormal	6 (25%)	34 (47%)	40 (41%)
Evolution from MDS→AML			
No	19 (79%)	46 (63%)	65 (67%)
Yes	5 (21%)	27 (37%)	32 (33%)

cMDS-LB (childhood MDS, with low blasts): <5% bone marrow blasts, MDS-LB (MDS, with low blasts): <5% bone marrow blasts, cMDS-IB (childhood MDS, with increased blasts): 5%–19% bone marrow blasts, MDS-IB-1 (MDS, with increased blasts-1): 5%–9% bone marrow blasts, MDS-IB-2 (MDS, with increased blasts-2): 9%–19% of blasts in the bone marrow, AML-MR: 20%–28% bone marrow blasts.

**FIGURE 1:**
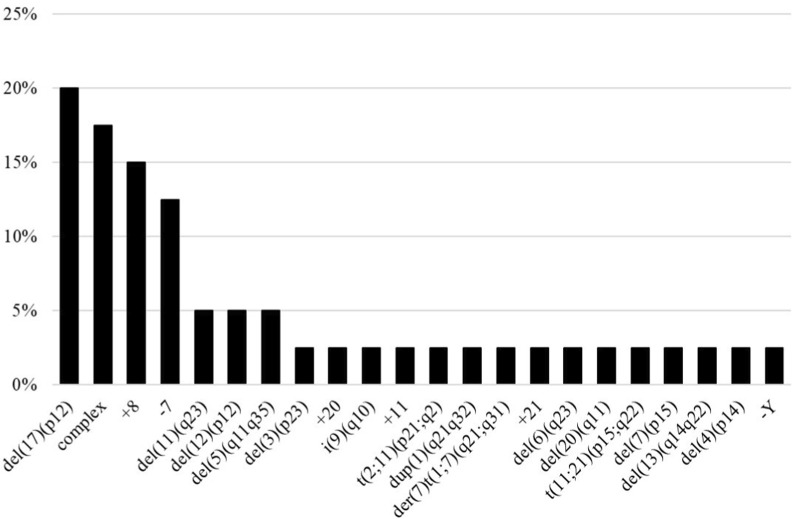
Frequency of chromosomal alterations in patients with MDS.

Cytogenetic analysis of MDS patients assessed for STAG2 expression revealed that 41% (40/97) exhibited abnormal karyotypes. Among these, clonal chromosomal abnormalities were identified in 25% (6/24) of pediatric patients and 46.5% (34/73) of adult patients. Further cytogenetic analysis revealed that, among patients with abnormal karyotypes, 50% (20/40) had structural alterations; 32.5% (13/40) had numerical alterations (aneuploidies); and 17.5% (7/40) presented both (six with complex karyotypes and one with a biclonal karyotype). The complex karyotype is defined as the presence of three or more chromosomal abnormalities in the same clone, while biclonal karyotypes represent unrelated clones that are detected simultaneously in a sample by G-band analysis. The distribution and frequency of chromosomal abnormalities in the MDS cohort are shown in Figure 1. The most frequent chromosomal alterations were deletion of the short arm of chromosome 17 [del (17p)], complex karyotypes, trisomy 8 (+8), and monosomy 7 (−7).

### 3.2 STAG2 expression profile in MDS patients

**FIGURE 2:**
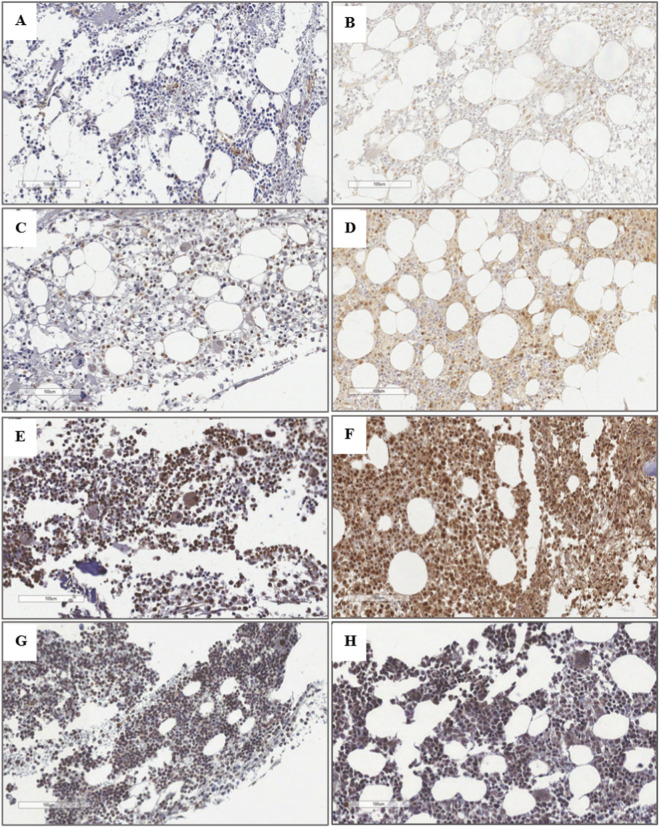
Immunohistochemical Analysis of STAG2 Expression Pattern in Bone Marrow Biopsies from MDS Patients and Neoplasia-Free Controls. **(A)** Pediatric MDS patient with an initial subtype, abnormal karyotype, hypocellular bone marrow, and low STAG2 expression. **(B)** Adult MDS patient with an advanced subtype, abnormal karyotype, hypercellular bone marrow, and low STAG2 expression. **(C)** Pediatric MDS patient with an initial subtype, normal karyotype, hypocellular bone marrow, and intermediate STAG2 expression. **(D)** Adult MDS patient with an initial subtype, normal karyotype, hypocellular bone marrow, and intermediate STAG2 expression. **(E)** Pediatric MDS patient with an initial subtype, normal karyotype, normocellular bone marrow, and high STAG2 expression. **(F)** Adult MDS patient with an initial subtype, normal karyotype, hypercellular bone marrow, and high STAG2 expression. **(G)** Neoplasia-free bone marrow biopsy from a pediatric patient showing high STAG2 expression. **(H)** Neoplasia-free bone marrow biopsy from an adult patient showing high STAG2 expression. Magnification: 100 µm.

The analysis of STAG2 expression in MDS patients revealed three distinct expression patterns: low, intermediate, and high, observed in both pediatric and adult cases. In contrast, control samples only exhibited a high expression pattern (Figure 2). Therefore, for further analysis, a high expression pattern was defined as the normal pattern, while low and intermediate expression levels were classified as reduced STAG2 expression.

Of all the patients analyzed, 67% (65/97) showed decreased STAG2 expression, with 51.5% (50/97) presenting an intermediate expression and 15.5% (15/97) presenting low expression. In turn, high expression was observed in 33% (32/97) of the patients. Stratification by age showed that 79% of pediatric patients had reduced STAG2 expression, with 21% (5/24) showing low expression and 58% (14/24) intermediate expression. High expression was observed in 21% (5/24) of pediatric patients. Concerning adults, reduced expression was identified in 63% (46/73), with 14% (10/73) showing low and 49% (36/73) intermediate expression, while 37% (27/73) retained high STAG2 expression (Table 2).

**TABLE 2:** STAG2 expression profile in pediatric and adult patients with MDS according to karyotype, disease subtype, and evolution toAML.

Clinical and cytogenetic features	STAG2 expression profile		
	Low	Intermediate	High
MDS patients	15,5% (15/97)	51,5% (50/97)	33% (32/97)
*Pediatric*	21% (5/24)	58% (14/24)	21% (5/24)
*Adult*	14% (10/73)	49% (36/73)	37% (27/73)
Karyotype			
Normal	12% (7/57)	53% (30/57)	35% (20/57)
Abnormal	20% (8/40)	50% (20/40)	30% (12/40)
*Pediatric*			
Normal	17% (3/18)	55% (10/18)	28% (5/18)
Abnormal	33% (2/6)	67% (4/6)	0% (0/6)
*Adult*			
Normal	10% (4/39)	51% (20/39)	39% (15/39)
Abnormal	18% (6/34)	47% (16/34)	35% (12/34)
Subtype			
Initial	17% (12/70)	50% (35/70)	33% (23/70)
Advanced	11% (3/27)	56% (15/27)	33% (9/27)
*Pediatric*			
Initial	23% (5/22)	54% (12/22)	23% (5/22)
Advanced	0% (0/2)	100% (2/2)	0% (0/2)
*Adult*			
Initial	14.5% (7/48)	48% (23/48)	37.5% (18/48)
Advanced	12% (3/25)	52% (13/25)	36% (9/25)
Evolution to AML			
No	14% (9/64)	55% (35/64)	31% (20/64)
Yes	18.2% (6/33)	45.5% (15/33)	36.3% (12/33)
*Pediatric*			
No	16.7% (3/18)	66.7% (12/18)	16.7% (3/18)
Yes	33% (2/6)	33% (2/6)	33% (2/6)
*Adult*			
No	13% (6/46)	50% (23/46)	37% (17/46)
Yes	15% (4/27)	48% (13/27)	37% (10/27)

Regarding the analysis of STAG2 expression according to karyotype, reduced expression of STAG2 was present in 65% (37/57) of patients with normal karyotypes and 70% (28/40) of patients with abnormal karyotypes. Within the pediatric subset of patients with abnormal karyotypes, 33% (2/6) had low STAG2 expression, while 67% (4/6) had intermediate expression. Notably, no pediatric patients with abnormal karyotypes demonstrated high STAG2 expression. In adults with abnormal karyotypes, 18% (6/34) exhibited low, 47% (16/34) intermediate, and 35% (12/34) high expression (Table 2). Reduced STAG2 expression was observed in 65% (13/20) of patients with structural changes, 69% (9/13) of patients with numerical changes (aneuploidy), and 86% (6/7) of patients with numerical and structural changes. Regarding specific chromosomal alterations, reduced STAG2 expression was observed in 86% (6/7) of patients with complex karyotypes, 83% (5/6) of those with trisomy 8, 62.5% (5/8) of those with del (17p), and 60% (3/5) of those with monosomy 7. Other cytogenetic alterations were observed in fewer than three patients and were therefore not analyzed separately.

Reduced STAG2 expression was found across both initial and advanced disease stages. Among patients with the initial subtypes, 67% (47/70) showed reduced STAG2 expression, including 17% (12/70) with low and 50% (35/70) with intermediate levels. In advanced stages, reduced expression was also present in 67% (18/27) of patients, with 11% (3/27) low and 55.5% (14/27) intermediate expression. The analyses by age group showed that 77% (17/22) of the pediatric patients classified in the initial subtype had reduced STAG2 expression, while in the advanced stages, all had reduced expression (2/2). In adult patients, 62.5% (30/48) of those classified in the initial subtypes showed decreased expression, while in advanced stages, 64% (16/25) of patients showed reduced STAG2 expression (Table 2).

The progression from MDS to AML was observed in 34% (33/97) of the patients. Reduced STAG2 expression was found in 69% (44/64) of those without disease evolution and 64% (21/33) of those who evolved to AML. Among pediatric patients who presented disease evolution, 33% (2/6) presented low expression, 33% (2/6) intermediate, and 33% (2/6) high expression. In adults who evolved to AML, 15% (4/27) had low expression, 48% (13/27) had intermediate, and 37% (10/27) had high expression of STAG2 (Table 2).

### 3.3 Bioinformatics analysis

**FIGURE 3:**
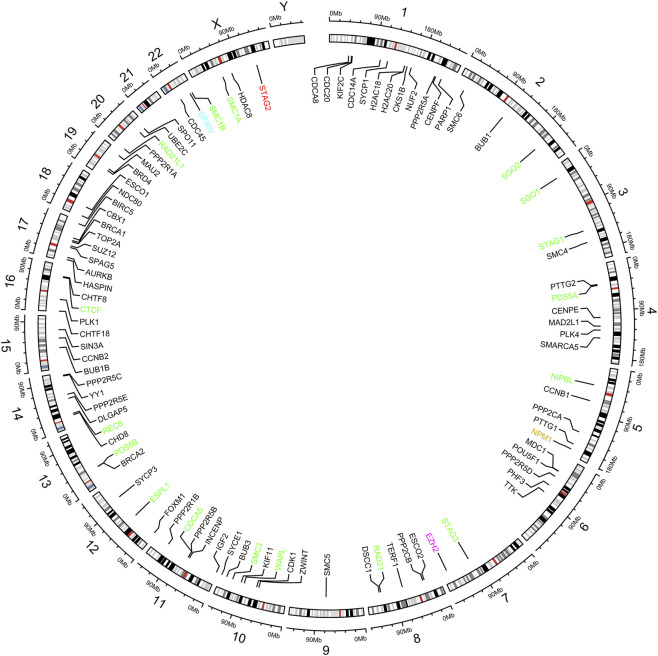
Circular diagram of the STAG2 gene interaction network across the human genome (Hg38). *STAG2* is highlighted in red. Direct interactions are shown in green, whereas all remaining gene connections represent indirect interactions. Genes associated with acute myeloid leukemia (AML) are indicated in blue, myelodysplastic neoplasms (MDS) in pink, and genes reported in both conditions (AML and MDS) in gold.

Independent STRING searches for *myelodysplastic syndrome* (MDS, DOID:0050908), *acute myeloid leukemia* (AML, DOID:9119), and *STAG2* retrieved from STRING 64, 83, and 94 interactors, respectively. A circular genomic plot illustrating the chromosomal distribution of genes encoding proteins directly or indirectly interacting with STAG2 showed that these interactions are present in all chromosomes except chromosome 21 and Y, which showed no STAG2-associated proteins. Clusters of interactions were observed on chromosomes 1, 17, 10, 14, and 4. Overlap analysis of STAG2 interactions with MDS and AML-associated proteins identified three proteins (EZH2, NPM1, STAG2) shared with the MDS PPI network, and two proteins (EP300 and NPM1) shared with the AML PPI network, with NPM1 being common to both conditions (Figure 3). However, STAG2 interactors included several proteins already associated with hematological cancers (Supplementary Tables S1, S2).

Functional enrichment analysis through GO (biological process, molecular function, and cellular component), KEGG, and Reactome demonstrated significant enrichment in terms related to the cell cycle, mitotic sister chromatid cohesion and segregation, mitotic checkpoint signalling, phosphatase activity, and microtubule binding. Enrichment analysis using the Transcription Factors PPIs database highlighted the top ten significantly enriched interactors, which included SMC3, HDAC2, RAD21, CTCF, TP53, MYC, POU5F1, MXI1, NOTCH1, and CREB1 (Supplementary Figure S1).

## 4 Discussion

Alterations in the cohesin complex have been recognized as a feature of myeloid neoplasms, including MDS, with *STAG2* representing the most frequently mutated component (Xu and Viny, 2024). Despite this, the protein expression profile of STAG2 in the bone marrow of MDS patients has not yet been investigated. In the present study, we demonstrated that neoplasia-free bone marrow samples exhibit a homogeneous high STAG2 expression pattern. This result suggests that this represents the physiological expression pattern in normal bone marrow. In contrast, MDS patients displayed a heterogeneous pattern with three distinct STAG2 expression profiles: high, intermediate, and low, with high expression observed in 33% of patients, whereas 67% showed either intermediate or low expression.

Given the rarity of pediatric MDS, most existing knowledge has been derived from studies conducted only in adult patients. However, the clinical and biological distinctions between age groups underscore the importance of studies that include pediatric and adult patients (Babcock et al., 2023; Schwartz et al., 2017; Jann and Tothova, 2021). In the present study, although the pediatric cohort was relatively small, the frequency of low STAG2 expression (21%) exceeded that observed in adult patients (14%). Interestingly, in both pediatric and adult cohorts, the frequency of low STAG2 expression was higher than the previously reported mutational rates, which range from 2.2% to 4.6% in pediatric patients and 5%–10% in adults (Bernard et al., 2022; Schwartz et al., 2017; Jann and Tothova, 2021; Haferlach, 2019; Kon et al., 2013; Shallis et al., 2018; Kozyra et al., 2021; Obenauer et al., 2016; West et al., 2022).

In solid tumors, such as melanoma and urothelial carcinoma, analyses of STAG2 expression through immunohistochemistry have also revealed a greater prevalence of reduced protein expression than would be expected from mutational rates alone (Gordon et al., 2022; Shen et al., 2016; Romero-Pérez et al., 2019). In MDS, in turn, although no previous studies have assessed STAG2 expression by immunohistochemistry, a large-scale analysis of RNA expression data from 183 cases in the TCGA repository demonstrated that approximately 10%–20% of patients exhibited reduced *STAG2* transcript levels (Thota et al., 2014). Consistent with our findings, this proportion was also higher than the reported mutational rates.

Moreover, Thota and colleagues (2014) showed that reduced *STAG2* expression could be associated with mutations in other cohesin complex components. However, diminished *STAG2* expression was also observed in the absence of detectable mutations in any cohesin complex genes (Thota et al., 2014). Taken together, these observations suggest the potential involvement of additional non-mutational regulatory mechanisms, such as transcriptional control, alternative splicing, epigenetic regulation, or post-translational modifications.

In this context, our bioinformatics analysis provides further support for the importance of assessing STAG2 expression at the protein level in MDS, as STAG2 interactors converge into three main mechanisms that are central to hematologic malignancies: (a) mitotic control and checkpoint dysfunction (AURKB, BUB1/BUB1B/BUB3, CDC20, PLK1, CDK1, and PTTG1) which contribute to uncontrolled proliferation and chromosomal instability; (b) chromatin and epigenetic regulation (EZH2, SUZ12, EP300, HDAC8, BRD4, PARP1, and the EP300/CREBBP axis) that disturb transcriptional programs and impair normal hematopoietic differentiation; (c) 3D genome architecture/cohesin pathway alterations (STAG2, RAD21, SMC1A, SMC3, and CTCF) that reshapes enhancer–promoter communication and lineage specification. Furthermore, seven of the top ten enriched transcription factors (TP53, MYC, NOTCH1, RAD21, SMC3, CTCF, and, in minor instance, HDAC2) have previously been implicated in hematologic malignancies (Bernard et al., 2022; Bersanelli et al., 2021; Shreve and Nazha, 2020; Khoury et al., 2022; Kuo and Dong, 2015).

Regarding cytogenetics, it is important to note that the canonical function of STAG2 is to ensure the cohesion of sister chromatids, particularly at the centromere, thereby maintaining chromosomal integrity during cell division. Early functional studies indicated that mutations in STAG2 often led to chromosomal abnormalities, primarily aneuploidies, in tumor cells (Solomon et al., 2014; Hill et al., 2016). However, subsequent research has shown that STAG2 mutations are not consistently associated with abnormal karyotypes across various neoplasms (Hill et al., 2016; Djos et al., 2013; Kim et al., 2016). In hematological malignancies, these alterations occur at comparable frequencies in patients with both normal and abnormal karyotypes, suggesting that chromosomal instability is not their inevitable consequence (Kon et al., 2013; Thota et al., 2014; Katamesh et al., 2023).

In the present study, reduced STAG2 expression was slightly more frequent in patients with abnormal karyotypes (70%) compared to those with normal ones (65%). Regarding specific cytogenetic alterations, +8 and complex karyotypes were associated with reduced STAG2 expression. These results are in accordance with prior studies linking +8 with STAG2 alterations (Thota et al., 2014; Toribio-Castelló et al., 2023). For instance, Thota and colleagues (2014) reported that patients with *STAG2* mutations had a higher frequency of +8 than those without such mutations (Thota et al., 2014). Additionally, Toribio-Castelló and colleagues. (2023) found that among patients with MDS and +8, 34.5% had *STAG2* mutations, and the co-occurrence of +8 and *STAG2* mutations was associated with worse outcomes (Toribio-Castelló et al., 2023).

The biological basis of the association between STAG2 and +8 remains unclear. Nonetheless, it is noteworthy that +8 is the most common chromosomal gain in MDS and represents an aneuploidy (Yan et al., 2021; Hosono, 2019). Furthermore, chromosome 8 harbors key genes such as the oncogene MYC (8q24.21) and RAD21 (8q24.11), another cohesin component that interacts with the STAG2 protein. Gaining extra copies of these genes can confer proliferative and survival advantages in hematopoietic clones (Jann et al., 2024). Then, the +8/STAG2-decreased clone may expand, accelerating progression from MDS to AML. Furthermore, it is plausible that the structural landscape and nuclear positioning of chromosome 8 make it more susceptible to segregation errors when cohesin function is impaired (Klaasen et al., 2022).

Complex karyotypes, by contrast, typically arise from pervasive genomic instability driven by defects in DNA repair pathways. STAG2 interacts closely with structures involved in DNA replication and repair. Among the interactors of STAG2 (Figure 3), for example, there is the architectural DNA-binding protein CTCF (CCCTC-binding factor), which functions in the cohesin complex to form chromatin loops and topologically associating domains (TADs). These structures organize the genome into functional compartments, bringing some regulatory elements together and keeping others apart (McGowan-Jordan et al., 2020; Del Moral-Morales et al., 2023).

By controlling looping and insulation, CTCF regulates when and where genes are expressed, participating in the regulation of DNA replication timing and protecting against chromosomal rearrangements by maintaining proper chromatin domain structure. CTCF establishes connections with a variety of epigenetic regulators, including histone- and DNA-demethylating enzymes, in addition to multiple long non-coding RNAs (lncRNAs) capable of guiding or recruiting it to specific genomic sites (Del Moral-Morales et al., 2023; Phipps and Dubrana, 2022). Consequently, reduced STAG2 expression could impair these processes and exacerbate chromosomal instability, thereby contributing to the development of complex karyotypes (Roig et al., 2014; Litwin et al., 2018; Phipps and Dubrana, 2022).

Functional studies employing loss-of-function models in both murine and human hematopoietic stem cells (HSCs) have demonstrated the essential role of cohesin complex components in regulating normal stem cell function (Kon et al., 2013). These models have shown that loss of expression of cohesin subunits, mainly STAG2, leads to disruption of HSC differentiation and self-renewal capacity that parallels those observed in MDS (Di Nardo et al., 2022; Viny and Levine, 2018; Viny et al., 2019; Awada et al., 2020; Bhattacharya et al., 2023). Consistently, our results show intermediate and low levels of STAG2 expression since the initial MDS subtypes, and with similar frequencies in advanced subtypes. These observations further suggest that STAG2 downregulation could be associated with the disruption in hematopoiesis characteristic of MDS happening as an early event in its pathogenesis.

Our expression study and bioinformatics analysis not only show that STAG2 and its interactors are involved in pathways that cooperate to hematopoiesis disruption but also uncover therapeutic opportunities (epigenetic drugs, aurora/PLK/CDK inhibitors, BET inhibitors, PARP inhibitors, and strategies targeting cohesin-related dependencies). PARP inhibitors, for example, are promising antitumor agents capable of increasing the sensitivity of cancer cells to treatments with DNA damage-inducing agents or inducing synthetic lethality in cells with deficiencies in the repair of double-strand breaks. Given the crucial role of the cohesin complex in DNA damage repair, PARP inhibitors could be particularly effective in treating myeloid neoplasms with alterations in cohesin complex components, especially STAG2 (Antony et al., 2021; Kontandreopoulou et al., 2021).

In summary, it is important to note that this is the first study to investigate STAG2 protein expression in bone marrow samples from patients with MDS. Therefore, further research is needed to validate these findings in independent cohorts and to better elucidate the role of aberrant STAG2 expression in the pathogenesis of the disease. Nevertheless, our results demonstrate that altered expression of this cohesin complex member may have important clinical and biological implications in MDS across both age groups. Moreover, immunohistochemistry is a cost-effective, rapid, and straightforward technique that can be easily implemented in clinical laboratories for diagnosis. As such, incorporating STAG2 expression analysis into diagnostic workflows for MDS may enable earlier identification of patients who are most likely to benefit from novel tailored therapeutic strategies.

## 5 Conclusion

Intermediate and low STAG2 expression levels were observed in bone marrow biopsies from MDS patients across all age groups, including those with early disease subtypes. This indicates that STAG2 downregulation may represent an early event in MDS pathogenesis. Furthermore, reduced STAG2 expression was closely associated with chromosomal instability, mainly in cases with trisomy 8 and complex karyotypes. Collectively, our findings suggest that reduced STAG2 protein expression plays a relevant role in MDS pathogenesis. Immunohistochemical assessment of STAG2 may be incorporated into the clinical workflows as a potential diagnostic and prognostic biomarker, as well as indicate therapeutic targets, particularly in association with complex karyotypes and trisomy 8.

## Data Availability

The original contributions presented in the study are included in the article/Supplementary Material, further inquiries can be directed to the corresponding author.

## Supplementary material

SUPPLEMENTARY FIGURE S1 Functional enrichment analysis of STAG2 and its interactions (first and second-level). The y-axis shows terms, and the x-axis describes the gene proportion (mapped against the total). Color represents categories, and circle size reflects statistical significance.
