# Supplementary figures and images for: Reduced STAG2 expression in myelodysplastic neoplasms and acute myeloid leukemia myelodysplasia-related: a potential biomarker associated with aneuploidy and disease progression
Source: Front Cell Dev Biol. 2026 Mar 20;14:1731983. doi: 10.3389/fcell.2026.1731983 (PMC13047209; doi:10.3389/fcell.2026.1731983)

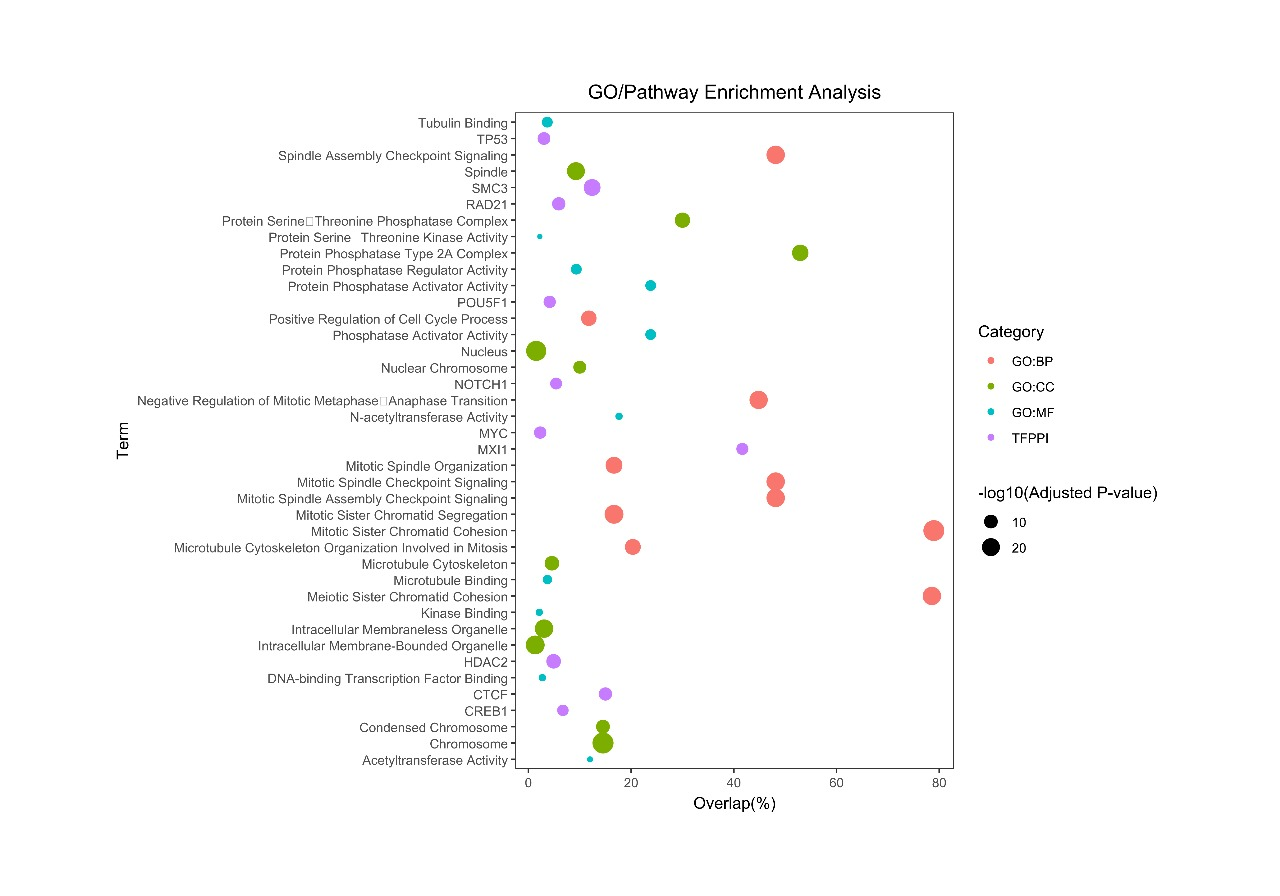

Supplement: Supplementary file 1 [file Image1.tif]
