# Supplementary material for: Reduced STAG2 expression in myelodysplastic neoplasms and acute myeloid leukemia myelodysplasia-related: a potential biomarker associated with aneuploidy and disease progression
Source: Front Cell Dev Biol. 2026 Mar 20;14:1731983. doi: 10.3389/fcell.2026.1731983 (PMC13047209; doi:10.3389/fcell.2026.1731983)
Supplement: Supplementary file 2 [file Table2.docx]

Supplementary Material

# Supplementary Tables

**Supplementary Table 1.** STAG2 direct and indirect interactors recovered by STRING.

**Supplementary Table 2.** Genes encoding proteins that interact with STAG2, their function and previous association with hematological cancers.

| **Gene** | **Main function** | **Evidence in hematological malignancies** | **References** |
| --- | --- | --- | --- |
| ***AURKB*** | Mitotic kinase (chromosome segregation) | Overexpressed/activated in AML and other hematologic neoplasms; aurora-B inhibitors evaluated preclinically/clinically | (Moreira-Nunes et al., 2020; Takeda et al., 2020; de Oliveira et al., 2022; Schwarz et al., 2022; Jin et al., 2024) |
| ***BIRC5* (Survivin)** | Inhibitor of apoptosis / mitotic regulator | Upregulated in AML/ALL/lymphomas; prognostic marker and therapeutic target | (Popek-Marciniec et al., 2023; Dunuwille et al., 2024; Yao et al., 2024; Kushwaha et al., 2025; Verdú-Bou et al., 2025) |
| ***BRD4*** | BET family chromatin reader, transcriptional co-activator | Required for leukemia transcriptional programs (MYC axis); BET inhibitors tested in AML/lymphoma | (Bauer et al., 2024; Zhang et al., 2024b; Chen et al., 2025a; Umphred-Wilson et al., 2025; Xu et al., 2025b) |
| ***BUB1* / *BUB1B* / *BUB3*** | Spindle assembly checkpoint proteins | Checkpoint dysfunction and altered expression reported in AML/MDS and other cancers | (Laberko et al., 2019; Kuttikrishnan et al., 2022; Richter-Pechańska et al., 2022; Cheng et al., 2023; Perlee et al., 2023; Akidan et al., 2025) |
| ***CDC20*** | APC/C activator (anaphase onset) | Frequently overexpressed in AML/MDS; linked to chromosomal instability and poor prognosis | (Liu et al., 2018; Chae et al., 2020; Wang et al., 2020; Castañeda-Partida et al., 2022; Zhou et al., 2024; Bahmei et al., 2025) |
| ***CDK1*** | G2–M cell cycle kinase | CDK1 activity supports AML proliferation; inhibitors show preclinical efficacy | (Ghelli Luserna di Rorà et al., 2019; Yang et al., 2021; Zhdanovskaya et al., 2022; Massacci et al., 2023; Manoochehrabadi et al., 2024) |
| ***CTCF*** | Genome architectural protein / insulator | Mutations and altered occupancy reported in AML/T-ALL; affects enhancer-promoter contacts in leukemia | (Gray et al., 2023; Mulet-Lazaro et al., 2024; Glushakow-Smith and Tothova, 2025; Hamamoto et al., 2025; Hyle et al., 2025) |
| ***EP300* (p300)** | Histone acetyltransferase / co-activator | Recurrently mutated/dysregulated in lymphomas and leukemias; epigenetic target | (Nicosia et al., 2023; Hou et al., 2024; Wu et al., 2024; Barua et al., 2025; Chen et al., 2025b; Shah et al., 2025) |
| ***EZH2*** | H3K27 methyltransferase (PRC2) | Gain-of-function mutations in follicular lymphoma/DLBCL; PRC2 alterations in myeloid neoplasms | (de Souza Fernandez et al., 2019; Sakhdari et al., 2022; Wang et al., 2022; Fang et al., 2024; Yu et al., 2025; Zhang et al., 2025) |
| ***HDAC8*** | Histone deacetylase (epigenetic regulator) | Implicated in AML/T-cell lymphomas; selective HDAC8 inhibitors show activity in preclinical leukemia models | (Spreafico et al., 2020; Wu et al., 2021; Zhang et al., 2021; Amin et al., 2023; Xu et al., 2025a) |
| ***NPM1*** | Nucleolar phosphoprotein (ribosome biogenesis) | One of the most frequent and defining mutations in AML (NPM1-mutant AML); diagnostic/prognostic marker | (Falini et al., 2020; Patel et al., 2020; Hindley et al., 2021; Morganti et al., 2022; Falini, 2023; Issa et al., 2023; Weinberg et al., 2023; Falini and Dillon, 2024) |
| ***PARP1*** | DNA damage sensor / repair enzyme | Overexpressed/active in some leukemias; PARP inhibitors under investigation in AML combinations | (Diamantopoulos et al., 2019; Gotoh et al., 2020; Padella et al., 2022; Okabe et al., 2023; Liu et al., 2024; Zhang et al., 2024a) |
| ***PLK1*** | Polo-like kinase (mitotic progression) | Overexpressed in AML; PLK1 inhibitors advanced into clinical trials for AML | (Min et al., 2019; Carr et al., 2021; Shah et al., 2023; Chen et al., 2024; Sheth et al., 2025) |
| ***PTTG1* (Securin)** | Regulates separase / sister chromatid separation | Overexpressed in many cancers including hematologic malignancies; associated with proliferation and poor prognosis | (Noll et al., 2015; Chen et al., 2018; Chateauvieux et al., 2020; Christiani et al., 2023) |
| ***RAD21*** | Cohesin complex subunit | Recurrent somatic mutations in AML/MDS; cohesin dysfunction implicated in myeloid neoplasia | (Stengel et al., 2021; Schedel et al., 2022; Boucher et al., 2024; Fischer et al., 2024; Jann et al., 2024; Laczko et al., 2024; Manola et al., 2024; Xu and Viny, 2024) |
| ***SMC1A*** | Cohesin complex subunit | Recurrently altered in AML/MDS; part of cohesin mutation spectrum affecting hematopoiesis | (Krug, 2010; Cessna et al., 2019; Zhao et al., 2019; Gadewal et al., 2020) |
| ***SMC3*** | Cohesin complex subunit | Mutations reported in myeloid malignancies; loss/alteration perturbs differentiation/self-renewal | (Kraft et al., 2019; Rivas et al., 2021; Arkoun et al., 2022; Khouri et al., 2025) |
| ***STAG2*** | Cohesin complex subunit | Frequently mutated in MDS/AML; STAG2 loss reshapes 3D chromatin and contributes to leukemogenesis | (Barwe et al., 2022; West et al., 2022; Boucher et al., 2024; Deb and Xiao, 2024; Fischer et al., 2024; Kimura et al., 2024; Xu and Viny, 2024; Khouri et al., 2025; Wong et al., 2025) |
| ***SUZ12*** | PRC2 subunit (epigenetic silencing) | PRC2 component alterations (including SUZ12) described in lymphomas and some leukemias; affects gene repression | (Broux et al., 2019; Kaito and Iwama, 2020; Schmied et al., 2020; Chai et al., 2024; Xiang et al., 2025) |
| ***YY1*** | Multifunctional transcription factor / chromatin regulator | Dysregulated in lymphomas/leukemias; involved in transcriptional programs and interaction with PRC2 (EZH2) | (Antonio-Andres et al., 2021; Jung et al., 2023; Noguera et al., 2023; Yang et al., 2024; He et al., 2025; Shimizu et al., 2025) |

# Supplementary Figures

**Supplementary Figure 1.** Functional enrichment analysis of STAG2 and its interactions (first- and second-level). The y-axis shows terms, and the x-axis describes the gene proportion (mapped against the total). Color represents categories, and circle size reflects statistical significance.

# References

Akidan, O., Petrovic, N., and Misir, S. (2025). mir-188‐5p emerges as an oncomir to promote chronic myeloid leukemia via upregulation of BUB3 and SUMO2. *Mol Biol Rep* 52, 269. doi: 10.1007/s11033-025-10359-9

Amin, S. A., Khatun, S., Gayen, S., Das, S., and Jha, T. (2023). Are inhibitors of histone deacetylase 8 (HDAC8) effective in hematological cancers especially acute myeloid leukemia (AML) and acute lymphoblastic leukemia (ALL)? *Eur J Med Chem* 258, 115594. doi: 10.1016/j.ejmech.2023.115594

Antonio-Andres, G., Jiménez-Hernandez, E., Estrada-Abreo, L. A., Garfias-Gómez, Y., Patino-Lopez, G., Juarez-Mendez, S., et al. (2021). Expression of YY1 in pro-B and T phenotypes correlation with poor survival in pediatric acute lymphoblastic leukemia. *Pediatr Hematol Oncol* 38, 456–470. doi: 10.1080/08880018.2020.1871139

Arkoun, B., Robert, E., Boudia, F., Mazzi, S., Dufour, V., Siret, A., et al. (2022). Stepwise GATA1 and SMC3 mutations alter megakaryocyte differentiation in a Down syndrome leukemia model. *Journal of Clinical Investigation* 132. doi: 10.1172/JCI156290

Bahmei, A., Fadakar, H., and Tamaddon, G. (2025). Deciphering the molecular landscape of acute myeloid leukemia initiation and relapse: a systems biology approach. *Medical Oncology* 42, 468. doi: 10.1007/s12032-025-03003-w

Barua, S., Murty, V. V., Iglesias, A., and Liao, J. (2025). A Novel EP300 Variant in an African American Girl With Global Developmental Delay and Leukemia. *Mol Genet Genomic Med* 13. doi: 10.1002/mgg3.70102

Barwe, S. P., Sebastian, A., Sidhu, I., Kolb, E. A., and Gopalakrishnapillai, A. (2022). Modeling Down Syndrome Myeloid Leukemia by Sequential Introduction of GATA1 and STAG2 Mutations in Induced Pluripotent Stem Cells with Trisomy 21. *Cells* 11, 628. doi: 10.3390/cells11040628

Bauer, K., Hauswirth, A., Gleixner, K. V., Greiner, G., Thaler, J., Bettelheim, P., et al. (2024). BRD4 degraders may effectively counteract therapeutic resistance of leukemic stem cells in AML and ALL. *Am J Hematol* 99, 1721–1731. doi: 10.1002/ajh.27385

Boucher, A., Murray, J., and Rao, S. (2024). Cohesin mutations in acute myeloid leukemia. *Leukemia* 38, 2318–2328. doi: 10.1038/s41375-024-02406-4

Broux, M., Prieto, C., Demeyer, S., Vanden Bempt, M., Alberti-Servera, L., Lodewijckx, I., et al. (2019). Suz12 inactivation cooperates with JAK3 mutant signaling in the development of T-cell acute lymphoblastic leukemia. *Blood* 134, 1323–1336. doi: 10.1182/blood.2019000015

Carr, R. M., Vorobyev, D., Lasho, T., Marks, D. L., Tolosa, E. J., Vedder, A., et al. (2021). RAS mutations drive proliferative chronic myelomonocytic leukemia via a KMT2A-PLK1 axis. *Nat Commun* 12, 2901. doi: 10.1038/s41467-021-23186-w

Castañeda-Partida, L., Ocadiz-Delgado, R., Sánchez-López, J. M., García-Villa, E., Peñaloza-González, J. G., Velázquez-Aviña, M. M., et al. (2022). Global expression profiling of CD10 + /CD19 + pre-B lymphoblasts from Hispanic B-ALL patients correlates with comparative TARGET database analysis. *Discover Oncology* 13, 28. doi: 10.1007/s12672-022-00480-7

Cessna, M. H., Paulraj, P., Hilton, B., Sadre-Bazzaz, K., Szankasi, P., Cluff, A., et al. (2019). Chronic myelomonocytic leukemia with ETV6-ABL1 rearrangement and SMC1A mutation. *Cancer Genet* 238, 31–36. doi: 10.1016/j.cancergen.2019.07.004

Chae, H.-D., Dutta, R., Tiu, B., Hoff, F. W., Accordi, B., Serafin, V., et al. (2020). RSK inhibitor BI-D1870 inhibits acute myeloid leukemia cell proliferation by targeting mitotic exit. *Oncotarget* 11, 2387–2403. doi: 10.18632/oncotarget.27630

Chai, J., Choudhuri, J., Gong, J. Z., Wang, Y., and Tian, X. (2024). Upregulation of Enhancer of Zeste Homolog 2 (EZH2) with Associated pERK Co-Expression and PRC2 Complex Protein SUZ12 Correlation in Adult T-Cell Leukemia/Lymphoma. *Cancers (Basel)* 16, 646. doi: 10.3390/cancers16030646

Chateauvieux, S., Gaigneaux, A., Gérard, D., Orsini, M., Morceau, F., Orlikova-Boyer, B., et al. (2020). Inflammation regulates long non-coding RNA-PTTG1-1:1 in myeloid leukemia. *Haematologica* 105, e280–e284. doi: 10.3324/haematol.2019.217281

Chen, M., Shen, C., Chen, Y., Chen, Z., Zhou, K., Chen, Y., et al. (2024). Metformin synergizes with gilteritinib in treating FLT3-mutated leukemia via targeting PLK1 signaling. *Cell Rep Med* 5, 101645. doi: 10.1016/j.xcrm.2024.101645

Chen, P.-Y., Tien, H.-J., Chen, S.-F., Horng, C.-T., Tang, H.-L., Jung, H.-L., et al. (2018). Response of Myeloid Leukemia Cells to Luteolin is Modulated by Differentially Expressed Pituitary Tumor-Transforming Gene 1 (PTTG1) Oncoprotein. *Int J Mol Sci* 19, 1173. doi: 10.3390/ijms19041173

Chen, Y., Huo, D., Meng, Y., Zhang, J., Huang, M., Luo, Q., et al. (2025a). BRD4 acts as a transcriptional repressor of RhoB to inhibit terminal erythropoiesis. *J Hematol Oncol* 18, 67. doi: 10.1186/s13045-025-01721-2

Chen, Y., Zhao, Y., Yao, M., Wang, Y., Ma, M., Yu, C., et al. (2025b). Concurrent inhibition of p300/CBP and FLT3 enhances cytotoxicity and overcomes resistance in acute myeloid leukemia. *Acta Pharmacol Sin* 46, 1390–1403. doi: 10.1038/s41401-025-01479-w

Cheng, C.-K., Yung, Y.-L., Chan, H.-Y., Leung, K.-T., Chan, K. Y. Y., Leung, A. W. K., et al. (2023). Deep genomic characterization highlights complexities and prognostic markers of pediatric acute myeloid leukemia. *Commun Biol* 6, 356. doi: 10.1038/s42003-023-04732-2

Christiani, E., Naumann, N., Weiss, C., Spiess, B., Kleiner, H., Fabarius, A., et al. (2023). Gene Expression Pattern of ESPL1, PTTG1 and PTTG1IP Can Potentially Predict Response to TKI First-Line Treatment of Patients with Newly Diagnosed CML. *Cancers (Basel)* 15, 2652. doi: 10.3390/cancers15092652

de Oliveira, F. M., Jamur, V. R., Merfort, L. W., Pozzo, A. R., and Mai, S. (2022). Three-dimensional nuclear telomere architecture and differential expression of aurora kinase genes in chronic myeloid leukemia to measure cell transformation. *BMC Cancer* 22, 1024. doi: 10.1186/s12885-022-10094-5

de Souza Fernandez, T., Fonseca Alvarenga, T., Almeida Antônio de Kós, E., Lamim Lovatel, V., Tavares, R. de C., da Costa, E. S., et al. (2019). Aberrant Expression of  EZH2 in Pediatric Patients with Myelodysplastic Syndrome: A Potential Biomarker of Leukemic Evolution. *Biomed Res Int* 2019, 1–9. doi: 10.1155/2019/3176565

Deb, P. Q., and Xiao, W. (2024). “Ring-form” megakaryocytic dysplasia in *STAG2* -mutated myelodysplastic neoplasm. *Blood* 143, 2218–2218. doi: 10.1182/blood.2024024069

Diamantopoulos, P. T., Kontandreopoulou, C.-N., Symeonidis, A., Kotsianidis, I., Pappa, V., Galanopoulos, A., et al. (2019). Bone marrow PARP1 mRNA levels predict response to treatment with 5-azacytidine in patients with myelodysplastic syndrome. *Ann Hematol* 98, 1383–1392. doi: 10.1007/s00277-019-03650-w

Dunuwille, W., Wilson, W. C., Bjeije, H., Issa, N., Han, W., Parsons, T. M., et al. (2024). BIRC5 upregulation enhances DNMT3A-mutant T-ALL cell survival and pathogenesis. *Blood Neoplasia* 1, 100040. doi: 10.1016/j.bneo.2024.100040

Falini, B. (2023). NPM1‐mutated acute myeloid leukemia: New pathogenetic and therapeutic insights and open questions. *Am J Hematol* 98, 1452–1464. doi: 10.1002/ajh.26989

Falini, B., Brunetti, L., Sportoletti, P., and Martelli, M. P. (2020). NPM1-mutated acute myeloid leukemia: from bench to bedside. *Blood* 136, 1707–1721. doi: 10.1182/blood.2019004226

Falini, B., and Dillon, R. (2024). Criteria for Diagnosis and Molecular Monitoring of NPM1-Mutated AML. *Blood Cancer Discov* 5, 8–20. doi: 10.1158/2643-3230.BCD-23-0144

Fang, J., Zhang, J., Zhu, L., Xin, X., and Hu, H. (2024). The epigenetic role of EZH2 in acute myeloid leukemia. *PeerJ* 12, e18656. doi: 10.7717/peerj.18656

Fischer, A., Hernández-Rodríguez, B., Mulet-Lazaro, R., Nuetzel, M., Hölzl, F., van Herk, S., et al. (2024). STAG2 mutations reshape the cohesin-structured spatial chromatin architecture to drive gene regulation in acute myeloid leukemia. *Cell Rep* 43, 114498. doi: 10.1016/j.celrep.2024.114498

Gadewal, N., Kumar, R., Aher, S., Gardane, A., Gaur, T., Varma, A. K., et al. (2020). miRNA‐mRNA Profiling Reveals Prognostic Impact of SMC1A Expression in Acute Myeloid Leukemia. *Oncology Research Featuring Preclinical and Clinical Cancer Therapeutics* 28, 321–330. doi: 10.3727/096504020X15816752427321

Ghelli Luserna di Rorà, A., Martinelli, G., and Simonetti, G. (2019). The balance between mitotic death and mitotic slippage in acute leukemia: a new therapeutic window? *J Hematol Oncol* 12, 123. doi: 10.1186/s13045-019-0808-4

Glushakow-Smith, S. G., and Tothova, Z. (2025). Role of Chromatin Looping Factors in Leukemia. *Annual Review of Pathology: Mechanisms of Disease*. doi: 10.1146/annurev-pathmechdis-051222-014420

Gotoh, N., Minato, Y., Saitoh, T., Takahashi, N., Kasamatsu, T., Souma, K., et al. (2020). PARP1 V762A polymorphism affects the prognosis of myelodysplastic syndromes. *Eur J Haematol* 104, 526–537. doi: 10.1111/ejh.13393

Gray, Z. H., Chakraborty, D., Duttweiler, R. R., Alekbaeva, G. D., Murphy, S. E., Chetal, K., et al. (2023). Epigenetic balance ensures mechanistic control of MLL amplification and rearrangement. *Cell* 186, 4528-4545.e18. doi: 10.1016/j.cell.2023.09.009

Hamamoto, K., Zhu, G., Lai, Q., Lesperance, J., Luo, H., Li, Y., et al. (2025). HoxBlinc lncRNA reprograms CTCF-independent TADs to drive leukemic transcription and HSC dysregulation in NUP98-rearranged leukemia. *Journal of Clinical Investigation* 135. doi: 10.1172/JCI184743

He, C., Xiong, Y., Zeng, Y., Feng, J., Yan, F., Zhang, M., et al. (2025). HDAC3-YY1-RAB5A axis remodels AML-supportive niche by modulating mitochondrial homeostasis in bone marrow stromal cells. *Cell Death Dis* 16, 498. doi: 10.1038/s41419-025-07777-9

Hindley, A., Catherwood, M. A., McMullin, M. F., and Mills, K. I. (2021). Significance of NPM1 Gene Mutations in AML. *Int J Mol Sci* 22, 10040. doi: 10.3390/ijms221810040

Hou, Z., Ren, Y., Zhang, X., Huang, D., Yan, F., Sun, W., et al. (2024). EP300-ZNF384 transactivates IL3RA to promote the progression of B-cell acute lymphoblastic leukemia. *Cell Communication and Signaling* 22, 211. doi: 10.1186/s12964-024-01596-9

Hyle, J., Qi, W., Djekidel, M. N., Rosikiewicz, W., Xu, B., and Li, C. (2025). Deciphering the role of RNA in regulating CTCF’s DNA binding affinity in leukemia cells. *Genome Biol* 26, 126. doi: 10.1186/s13059-025-03582-x

Issa, G. C., Aldoss, I., DiPersio, J., Cuglievan, B., Stone, R., Arellano, M., et al. (2023). The menin inhibitor revumenib in KMT2A-rearranged or NPM1-mutant leukaemia. *Nature* 615, 920–924. doi: 10.1038/s41586-023-05812-3

Jann, J.-C., Hergott, C. B., Winkler, M., Liu, Y., Braun, B., Charles, A., et al. (2024). Subunit-specific analysis of cohesin-mutant myeloid malignancies reveals distinct ontogeny and outcomes. *Leukemia* 38, 1992–2002. doi: 10.1038/s41375-024-02347-y

Jin, J., Hou, S., Yao, Y., Liu, M., Mao, L., Yang, M., et al. (2024). Phosphoproteomic Characterization and Kinase Signature Predict Response to Venetoclax Plus 3+7 Chemotherapy in Acute Myeloid Leukemia. *Adv Sci (Weinh)* 11, e2305885. doi: 10.1002/advs.202305885

Jung, M., Bui, I., and Bonavida, B. (2023). Role of YY1 in the Regulation of Anti-Apoptotic Gene Products in Drug-Resistant Cancer Cells. *Cancers (Basel)* 15, 4267. doi: 10.3390/cancers15174267

Kaito, S., and Iwama, A. (2020). Pathogenic Impacts of Dysregulated Polycomb Repressive Complex Function in Hematological Malignancies. *Int J Mol Sci* 22, 74. doi: 10.3390/ijms22010074

Khouri, M. R., Wang, B., Pearson, L. K., Gillis‐Smith, A. J., Suzuki, S., Hutchinson, L. M., et al. (2025). Characteristics and clinical outcomes of patients with myeloid malignancies and cohesin mutations. *Cancer* 131. doi: 10.1002/cncr.35846

Kimura, S., Park, C. S., Montefiori, L. E., Iacobucci, I., Pölönen, P., Gao, Q., et al. (2024). Biologic and Clinical Analysis of Childhood Gamma Delta T-ALL Identifies *LMO2/STAG2* Rearrangements as Extremely High Risk. *Cancer Discov* 14, 1838–1859. doi: 10.1158/2159-8290.CD-23-1452

Kraft, B., Lombard, J., Kirsch, M., Wuchter, P., Bugert, P., Hielscher, T., et al. (2019). SMC3 protein levels impact on karyotype and outcome in acute myeloid leukemia. *Leukemia* 33, 795–799. doi: 10.1038/s41375-018-0287-6

Krug (2010). Low SMC1A protein expression predicts poor survival in acute myeloid leukemia. *Oncol Rep* 24. doi: 10.3892/or_00000827

Kushwaha, A. C., Mrunalini, B., Malhotra, P., Karmakar, S., and Roy Choudhury, S. (2025). Bone-Marrow-Targeted Nanocomposite Abrogates C-Myb-Survivin Cross Talk in MLL-AF9-Rearranged Acute Myeloid Leukemia in *In Vitro* and *In Vivo* Patient-Derived Xenograft Models. *ACS Appl Mater Interfaces* 17, 711–724. doi: 10.1021/acsami.4c18737

Kuttikrishnan, S., Masoodi, T., Sher, G., Bhat, A. A., Patil, K., El-Elimat, T., et al. (2022). Bioinformatics Analysis Reveals FOXM1/BUB1B Signaling Pathway as a Key Target of Neosetophomone B in Human Leukemic Cells: A Gene Network-Based Microarray Analysis. *Front Oncol* 12. doi: 10.3389/fonc.2022.929996

Laberko, A., Balashov, D., Deripapa, E., Soldatkina, O., Raikina, E., Maschan, A., et al. (2019). Hematopoietic stem cell transplantation in a patient with type 1 mosaic variegated aneuploidy syndrome. *Orphanet J Rare Dis* 14, 97. doi: 10.1186/s13023-019-1073-x

Laczko, D., Poveda-Rogers, C., Matthews, A. H., Snaith, O., Luger, S., Bagg, A., et al. (2024). RAD21 mutations in acute myeloid leukemia. *Leuk Lymphoma* 65, 958–964. doi: 10.1080/10428194.2024.2328233

Liu, X., Zhang, F., Zhang, Y., Li, X., Chen, C., Zhou, M., et al. (2018). PPM1K Regulates Hematopoiesis and Leukemogenesis through CDC20-Mediated Ubiquitination of MEIS1 and p21. *Cell Rep* 23, 1461–1475. doi: 10.1016/j.celrep.2018.03.140

Liu, Z. S., Sinha, S., Bannister, M., Song, A., Arriaga-Gomez, E., McKeeken, A. J., et al. (2024). R-Loop Accumulation in Spliceosome Mutant Leukemias Confers Sensitivity to PARP1 Inhibition by Triggering Transcription–Replication Conflicts. *Cancer Res* 84, 577–597. doi: 10.1158/0008-5472.CAN-23-3239

Manola, K. N., Zachaki, S., Kakosaiou, K., Ioannidou, A., Kalomoiraki, M., and Rampias, T. (2024). Cohesin RAD21 Gene Promoter Methylation in Patients with Acute Myeloid Leukemia. *Life* 14, 1311. doi: 10.3390/life14101311

Manoochehrabadi, S., Talebi, M., Pashaiefar, H., Ghafouri-Fard, S., Vaezi, M., Omrani, M. D., et al. (2024). Upregulation of lnc-FOXD2-AS1, CDC45, and CDK1 in patients with primary non-M3 AML is associated with a worse prognosis. *Blood Res* 59, 4. doi: 10.1007/s44313-024-00002-0

Massacci, G., Venafra, V., Latini, S., Bica, V., Pugliese, G. M., Graziosi, S., et al. (2023). A key role of the WEE1-CDK1 axis in mediating TKI-therapy resistance in FLT3-ITD positive acute myeloid leukemia patients. *Leukemia* 37, 288–297. doi: 10.1038/s41375-022-01785-w

Min, K. Il, Park, S., Shin, S.-H., Kwon, Y.-R., Kim, H.-J., and Kim, Y. J. (2019). Enhanced polo-like kinase 1 expression in myelodysplastic syndromes. *Blood Res* 54, 102–107. doi: 10.5045/br.2019.54.2.102

Moreira-Nunes, C. A., Mesquita, F. P., Portilho, A. J. de S., Mello Júnior, F. A. R., Maués, J. H. da S., Pantoja, L. da C., et al. (2020). Targeting aurora kinases as a potential prognostic and therapeutical biomarkers in pediatric acute lymphoblastic leukaemia. *Sci Rep* 10, 21272. doi: 10.1038/s41598-020-78024-8

Morganti, C., Ito, K., Yanase, C., Verma, A., Teruya‐Feldstein, J., and Ito, K. (2022). *NPM1* ablation induces HSC aging and inflammation to develop myelodysplastic syndrome exacerbated by *p53* loss. *EMBO Rep* 23. doi: 10.15252/embr.202154262

Mulet-Lazaro, R., van Herk, S., Nuetzel, M., Sijs-Szabo, A., Díaz, N., Kelly, K., et al. (2024). Epigenetic alterations affecting hematopoietic regulatory networks as drivers of mixed myeloid/lymphoid leukemia. *Nat Commun* 15, 5693. doi: 10.1038/s41467-024-49811-y

Nicosia, L., Spencer, G. J., Brooks, N., Amaral, F. M. R., Basma, N. J., Chadwick, J. A., et al. (2023). Therapeutic targeting of EP300/CBP by bromodomain inhibition in hematologic malignancies. *Cancer Cell* 41, 2136-2153.e13. doi: 10.1016/j.ccell.2023.11.001

Noguera, N. I., Travaglini, S., Scalea, S., Catalanotto, C., Reale, A., Zampieri, M., et al. (2023). YY1 Knockdown Relieves the Differentiation Block and Restores Apoptosis in AML Cells. *Cancers (Basel)* 15, 4010. doi: 10.3390/cancers15154010

Noll, J. E., Vandyke, K., Hewett, D. R., Mrozik, K. M., Bala, R. J., Williams, S. A., et al. (2015). PTTG1 expression is associated with hyperproliferative disease and poor prognosis in multiple myeloma. *J Hematol Oncol* 8, 106. doi: 10.1186/s13045-015-0209-2

Okabe, S., Tanaka, Y., Moriyama, M., and Gotoh, A. (2023). WEE1 and PARP-1 play critical roles in myelodysplastic syndrome and acute myeloid leukemia treatment. *Cancer Cell Int* 23, 128. doi: 10.1186/s12935-023-02961-3

Padella, A., Ghelli Luserna Di Rorà, A., Marconi, G., Ghetti, M., Martinelli, G., and Simonetti, G. (2022). Targeting PARP proteins in acute leukemia: DNA damage response inhibition and therapeutic strategies. *J Hematol Oncol* 15, 10. doi: 10.1186/s13045-022-01228-0

Patel, S. S., Kluk, M. J., and Weinberg, O. K. (2020). NPM1 Biology in Myeloid Neoplasia. *Curr Hematol Malig Rep* 15, 350–359. doi: 10.1007/s11899-020-00592-3

Perlee, S., Kikuchi, S., Nakadai, T., Masuda, T., Ohtsuki, S., Matsumoto, M., et al. (2023). SETD1A function in leukemia is mediated through interaction with mitotic regulators BuGZ/BUB3. *EMBO Rep* 24. doi: 10.15252/embr.202357108

Popek-Marciniec, S., Zmorzyński, S., Koczkodaj, D., Marciniec, M., Wąsik-Szczepanek, E., Karczmarczyk, A., et al. (2023). CCL3 as Possible Negative Prognostic Factor in Chronic Lymphocytic Leukemia. *Acta Haematol* 146, 277–286. doi: 10.1159/000526397

Richter-Pechańska, P., Kunz, J. B., Rausch, T., Erarslan-Uysal, B., Bornhauser, B., Frismantas, V., et al. (2022). Pediatric T-ALL type-1 and type-2 relapses develop along distinct pathways of clonal evolution. *Leukemia* 36, 1759–1768. doi: 10.1038/s41375-022-01587-0

Rivas, M. A., Meydan, C., Chin, C. R., Challman, M. F., Kim, D., Bhinder, B., et al. (2021). Smc3 dosage regulates B cell transit through germinal centers and restricts their malignant transformation. *Nat Immunol* 22, 240–253. doi: 10.1038/s41590-020-00827-8

Sakhdari, A., Class, C., Montalban-Bravo, G., Sasaki, K., Bueso-Ramos, C. E., Patel, K. P., et al. (2022). Immunohistochemical loss of enhancer of Zeste Homolog 2 (EZH2) protein expression correlates with EZH2 alterations and portends a worse outcome in myelodysplastic syndromes. *Modern Pathology* 35, 1212–1219. doi: 10.1038/s41379-022-01074-y

Schedel, A., Friedrich, U. A., Morcos, M. N. F., Wagener, R., Mehtonen, J., Watrin, T., et al. (2022). Recurrent Germline Variant in RAD21 Predisposes Children to Lymphoblastic Leukemia or Lymphoma. *Int J Mol Sci* 23, 5174. doi: 10.3390/ijms23095174

Schmied, L., Olofsen, P. A., Lundberg, P., Tzankov, A., Kleber, M., Halter, J., et al. (2020). Secondary CNL after SAA reveals insights in leukemic transformation of bone marrow failure syndromes. *Blood Adv* 4, 5540–5546. doi: 10.1182/bloodadvances.2020001541

Schwarz, A., Roeder, I., and Seifert, M. (2022). Comparative Gene Expression Analysis Reveals Similarities and Differences of Chronic Myeloid Leukemia Phases. *Cancers (Basel)* 14. doi: 10.3390/cancers14010256

Shah, K., Nasimian, A., Ahmed, M., Al Ashiri, L., Denison, L., Sime, W., et al. (2023). PLK1 as a cooperating partner for BCL2-mediated antiapoptotic program in leukemia. *Blood Cancer J* 13, 139. doi: 10.1038/s41408-023-00914-7

Shah, V., Giotopoulos, G., Osaki, H., Meyerhöfer, M., Meduri, E., Gallego-Crespo, A., et al. (2025). Acute resistance to BET inhibitors remodels compensatory transcriptional programs via p300 coactivation. *Blood* 145, 748–764. doi: 10.1182/blood.2022019306

Sheth, A. S., Chan, K.-K., Liu, S., Wan, J., Angus, S. P., Rhodes, S. D., et al. (2025). PLK1 Inhibition Induces Synthetic Lethality in Fanconi Anemia Pathway–Deficient Acute Myeloid Leukemia. *Cancer Research Communications* 5, 648–667. doi: 10.1158/2767-9764.CRC-24-0260

Shimizu, T., Shindo, T., Ogawa, H., Teranaka, K., Watanabe, A., and Takaori‐Kondo, A. (2025). Upregulation of YY1/EZH2 and MLH1 as Therapeutic Targets for Adult T‐Cell Leukemia/Lymphoma. *Cancer Sci* 116, 2163–2175. doi: 10.1111/cas.70095

Spreafico, M., Gruszka, A. M., Valli, D., Mazzola, M., Deflorian, G., Quintè, A., et al. (2020). HDAC8: A Promising Therapeutic Target for Acute Myeloid Leukemia. *Front Cell Dev Biol* 8. doi: 10.3389/fcell.2020.00844

Stengel, A., Baer, C., Walter, W., Meggendorfer, M., Kern, W., Haferlach, T., et al. (2021). Mutational patterns and their correlation to CHIP-related mutations and age in hematological malignancies. *Blood Adv* 5, 4426–4434. doi: 10.1182/bloodadvances.2021004668

Takeda, T., Tsubaki, M., Genno, S., Nemoto, C., Onishi, Y., Yamamoto, Y., et al. (2020). AT9283 exhibits antiproliferative effect on tyrosine kinase inhibitor‑sensitive and ‑resistant chronic myeloid leukemia cells by inhibition of Aurora A and Aurora B. *Oncol Rep* 44, 2211–2218. doi: 10.3892/or.2020.7739

Umphred-Wilson, K., Ratnayake, S., Tang, Q., Wang, R., Chaudhary, S. G., Ballachanda, D. N., et al. (2025). The ESCRT protein CHMP5 promotes T cell leukemia by enabling BRD4-p300-dependent transcription. *Nat Commun* 16, 4133. doi: 10.1038/s41467-025-59504-9

Verdú-Bou, M., Baptista, M. J., Ribeiro, M. L., Méndez-López, A., Profitós-Pelejà, N., Frontzek, F., et al. (2025). The role of miR-150-5p/E2F3/survivin axis in the pathogenesis of plasmablastic lymphoma and its therapeutic potential. *Blood Adv* 9, 2953–2967. doi: 10.1182/bloodadvances.2025016180

Wang, J., He, N., Wang, R., Tian, T., Han, F., Zhong, C., et al. (2020). Analysis of TET2 and EZH2 gene functions in chromosome instability in acute myeloid leukemia. *Sci Rep* 10, 2706. doi: 10.1038/s41598-020-59365-w

Wang, J., Yu, X., Gong, W., Liu, X., Park, K.-S., Ma, A., et al. (2022). EZH2 noncanonically binds cMyc and p300 through a cryptic transactivation domain to mediate gene activation and promote oncogenesis. *Nat Cell Biol* 24, 384–399. doi: 10.1038/s41556-022-00850-x

Weinberg, O. K., Porwit, A., Orazi, A., Hasserjian, R. P., Foucar, K., Duncavage, E. J., et al. (2023). The International Consensus Classification of acute myeloid leukemia. *Virchows Archiv* 482, 27–37. doi: 10.1007/s00428-022-03430-4

West, R. R., Calvo, K. R., Embree, L. J., Wang, W., Tuschong, L. M., Bauer, T. R., et al. (2022). *ASXL1* and *STAG2* are common mutations in GATA2 deficiency patients with bone marrow disease and myelodysplastic syndrome. *Blood Adv* 6, 793–807. doi: 10.1182/bloodadvances.2021005065

Wong, W. J., Zon, R. L., Gibson, C. J., Ho, C., Pozdnyakova, O., Neuberg, D., et al. (2025). Somatic mutations in STAG2 are associated with separated megakaryocyte nuclear lobes in myelodysplastic syndromes. *Blood Adv* 9, 5283–5288. doi: 10.1182/bloodadvances.2025016897

Wu, J., Zhang, L., Feng, Y., Khadka, B., Fang, Z., and Liu, J. (2021). HDAC8 promotes daunorubicin resistance of human acute myeloid leukemia cells via regulation of IL-6 and IL-8. *Biol Chem* 402, 461–468. doi: 10.1515/hsz-2020-0196

Wu, X., Yang, X., Chen, Y., Zhang, Z., and XinhongYang (2024). A Rare Case Report of B-Cell Acute Lymphoblastic Leukemia with EP300::ZNF384 Fusion Gene. *Clin Lab* 70. doi: 10.7754/Clin.Lab.2023.231226

Xiang, L., Lin, X., and Wu, Y. (2025). LINC-PINT suppresses the progression of acute myeloid leukemia via miR-767-5p/SUZ12-mediated JAK/STAT signaling pathway. *Cytokine* 188, 156883. doi: 10.1016/j.cyto.2025.156883

Xu, G., Wang, F., Chen, M., Gao, W., Liu, Y., Zhu, J., et al. (2025a). Targeting HDAC8 sensitizes tyrosine kinase inhibitors in the elimination of B-cell acute lymphoblastic leukemia cells through degradation of HIF-1α. *Leukemia*. doi: 10.1038/s41375-025-02749-6

Xu, J. J., and Viny, A. D. (2024). Chromatin organization in myelodysplastic syndrome. *Exp Hematol* 134, 104216. doi: 10.1016/j.exphem.2024.104216

Xu, Y., Yang, H., Li, Y., Qi, Y., Zhao, F., Hong, Y., et al. (2025b). Discovery of highly potent dual GSPT1/BRD4 degraders with anti-AML activity. *Eur J Med Chem* 288, 117381. doi: 10.1016/j.ejmech.2025.117381

Yang, F., Zhou, H., Luo, P., Jia, L., Hou, M., Huang, J., et al. (2024). Celastrol induces DNA damage and cell death in BCR-ABL T315I-mutant CML by targeting YY1 and HMCES. *Phytomedicine* 134, 155937. doi: 10.1016/j.phymed.2024.155937

Yang, Y., Dai, Y., Yang, X., Wu, S., and Wang, Y. (2021). DNMT3A Mutation-Induced CDK1 Overexpression Promotes Leukemogenesis by Modulating the Interaction between EZH2 and DNMT3A. *Biomolecules* 11, 781. doi: 10.3390/biom11060781

Yao, M., Jiang, X., Xiao, F., Lv, X., Sheng, M., Xing, W., et al. (2024). Targeting BIRC5 as a therapeutic approach to overcome ASXL1-associated decitabine resistance. *Cancer Lett* 593, 216949. doi: 10.1016/j.canlet.2024.216949

Yu, H., Hong, J., Shin, D.-Y., and Lee, C.-H. (2025). The role of ASXL1, SRSF2, and EZH2 mutations in chromatin dysregulation of myelodysplastic neoplasia and acute myeloid leukemia. *Leukemia* 39, 2329–2339. doi: 10.1038/s41375-025-02657-9

Zhang, F., Sun, J., Zhang, L., Li, R., Wang, Y., Geng, H., et al. (2024a). PARP inhibition leads to synthetic lethality with key splicing-factor mutations in myelodysplastic syndromes. *Br J Cancer* 131, 231–242. doi: 10.1038/s41416-024-02729-0

Zhang, L., Nguyen, L. X. T., Chen, Y.-C., Wu, D., Cook, G. J., Hoang, D. H., et al. (2021). Targeting miR-126 in inv(16) acute myeloid leukemia inhibits leukemia development and leukemia stem cell maintenance. *Nat Commun* 12, 6154. doi: 10.1038/s41467-021-26420-7

Zhang, Q., Luo, Y., Ye, L., Wang, Y., Wang, L., Yang, W., et al. (2025). EZH2 inhibition induces pyroptosis via RHA-mediated S100A9 overexpression in myelodysplastic syndromes. *Exp Hematol Oncol* 14, 9. doi: 10.1186/s40164-025-00600-3

Zhang, Y., Lou, J., Liu, Y., Jin, P., Tan, Y., Song, H., et al. (2024b). Phase separation of PML/RARα and BRD4 coassembled microspeckles governs transcriptional dysregulation in acute promyelocytic leukemia. *Proceedings of the National Academy of Sciences* 121. doi: 10.1073/pnas.2406519121

Zhao, C., Wang, S., Zhao, Y., Du, F., Wang, W., Lv, P., et al. (2019). Long noncoding RNA NEAT1 modulates cell proliferation and apoptosis by regulating miR‐23a‐3p/SMC1A in acute myeloid leukemia. *J Cell Physiol* 234, 6161–6172. doi: 10.1002/jcp.27393

Zhdanovskaya, N., Lazzari, S., Caprioglio, D., Firrincieli, M., Maioli, C., Pace, E., et al. (2022). Identification of a Novel Curcumin Derivative Influencing Notch Pathway and DNA Damage as a Potential Therapeutic Agent in T-ALL. *Cancers (Basel)* 14, 5772. doi: 10.3390/cancers14235772

Zhou, Q., Li, Z., Meng, L., Wang, Y., Ashaq, M. S., Li, Y., et al. (2024). Identification of ubiquitination-related hub genes in chronic myeloid leukemia cell by bioinformatics analysis. *J Cancer* 15, 3750–3759. doi: 10.7150/jca.96405
